# Identification of crucial drought-tolerant genes of barley through comparative transcriptomic analysis and yeast-based stress assay

**DOI:** 10.3389/fgene.2024.1524118

**Published:** 2024-12-09

**Authors:** Xiaoyan He, Congjun Su, Xinyi Zhang, Zhaoxia Shi, Yanjie Wang, Huandi Peng, Shuying Fang, Xinyu Chen, Huayan Yin, Jianbin Zeng, Ping Mu

**Affiliations:** College of Agronomy, Qingdao Agricultural University, Qingdao, China

**Keywords:** barley, drought, transcriptome sequencing, metabolic pathway, yeast-based stress assay

## Abstract

Drought is a persistent and serious threat to crop yield and quality. The identification and functional characterization of drought tolerance-related genes is thus vital for efforts to support the genetic improvement of drought-tolerant crops. Barley is highly adaptable and renowned for its robust stress resistance, making it an ideal subject for efforts to explore genes related to drought tolerance. In this study, two barley materials with different drought tolerance were subjected to soil drought treatment, including a variety with strong drought tolerance (Hindmarsh) and a genotype with weaker drought tolerance (XZ5). Transcriptomic sequencing data from the aboveground parts of these plants led to the identification of 1,206 differentially expressed genes associated with drought tolerance. These genes were upregulated in Hindmarsh following drought stress exposure but downregulated or unchanged in XZ5 under these same conditions, or were unchanged in Hindmarsh but downregulated in XZ5. Pathway enrichment analyses suggested that these genes are most closely associated with defense responses, signal recognition, photosynthesis, and the biosynthesis of various secondary metabolites. Using protein-protein interaction networks, the ankyrin repeat domain-containing protein 17-like isoform X2 was predicted to impact other drought tolerance-related protein targets in Hindmarsh. In MapMan metabolic pathway analyses, genes found to be associated with the maintenance of drought tolerance in Hindmarsh under adverse conditions were predicted to include genes involved in the abscisic acid, cytokinin, and gibberellin phytohormone signaling pathways, genes associated with redox homeostasis related to ascorbate and glutathione S-transferase, transporters including ABC and AAAP, transcription factors such as AP2/ERF and bHLH, the heat shock proteins HSP60 and HSP70, and the sucrose non-fermenting-1-related protein kinase. Heterologous *HvSnRK2* (one of the identified genes, which encodes the sucrose non-fermenting-1-related protein kinase) gene expression in yeast conferred significant drought tolerance, highlighting the functional importance of this gene as one linked with drought tolerance. This study revealed the drought tolerance mechanism of Hindmarsh by comparing transcriptomes while also providing a set of candidate genes for genetic efforts to improve drought tolerance in this and other crop species.

## 1 Introduction

Water comprises roughly 80%–95% of fresh plant body biomass, serving as an essential medium for the physiological processes that support growth, development, and metabolic activity (Eziz et al., 2017). The photosynthetic products generated within plant leaves form the material basis for plant growth, with the net rate of photosynthesis thus directly reflecting overall material productivity per leaf area. Given its importance, photosynthesis is profoundly sensitive to drought conditions. Indeed, drought is estimated to result in greater annual losses than those attributable to all known pathogens in terms of crop yields (Gupta et al., 2020). Drought is thus the most prominent environmental stressor to which plants are exposed, particularly in areas prone to drought conditions (Dietz et al., 2021), making it the single most important threat to future global food security (He et al., 2019). To adapt to drought conditions, plants have evolved a range of physiological, cellular, metabolic, and molecular responses that can support their survival (Seleiman et al., 2021). Under conditions of drought, plants detect water stress-related signals and produce a range of signaling molecules including abscisic acid (ABA), reactive oxygen species (ROS), and Ca^2+^, that can engage signal transduction pathways that indirectly or directly alter plant morphology and physiology (Liu et al., 2022). Drought stress signals can indirectly induce downstream gene expression. Plant physiology can be influenced by several metabolism-related genes, including those encoding aquaporins (AQPs) and late embryogenesis abundant (LEA) proteins (Jia et al., 2022; Wu et al., 2022). Factors responsible for the maintaining of redox homeostasis, including glutathione S-transferase and ascorbate, can directly react with ROS or serve as substrates for enzymes important for ROS scavenging (Dusart et al., 2019). Plant physiology and morphology can also be influenced by proteins such as calcium-dependent protein kinases (SnRKs), mitogen-activated protein kinases (MAPKs), HD-zip/bZIP, AP2/ERF, NAC, MYB, bHLH, and WRKY, which can modulate signal transduction or serve as transcription factors that modulate downstream gene expression to enable better plant survival under arid conditions (Yang et al., 2021). While there have been some prior studies of particular genes and signaling pathways associated with drought stress, the complex mechanisms that govern how plants respond to drought conditions remain incompletely characterized. There is thus a clear need to better define additional molecular elements related to drought stress responses and characterize valuable gene targets for the improvement of plant drought tolerance.

Increasingly advanced next-generation sequencing technologies have enabled routine high-throughput RNA sequencing (Wang et al., 2009). Large-scale transcriptomic analyses can achieve greater coverage depth in less time than older approaches, thereby supporting metabolic pathway analyses, evolutionary genomics studies, comparative transcriptomics, and gene discovery efforts (Ozsolak and Milos, 2011). Comparing transcriptomes associated with different levels of stress tolerance is an effective means of analyzing the mechanistic basis for stress responses in various crops. For instance, a study of the transcriptomes of two peanut varieties with different levels of drought tolerance, NH5 (tolerant) and FH18 (sensitive), led to the identification of the activation of ABA and salicylic acid (SA) signaling within peanut plants exposed to drought stress. The enhanced drought tolerance of the NH5 variety was ultimately attributed to differences in the expression of genes related to ROS neutralization, osmotic adjustment, and cell wall fortification (Jiang et al., 2021). To clarify the molecular basis for soybean drought tolerance, comparative transcriptomic analyses were performed using seedlings from the drought-tolerant “Jindou 21” and drought-sensitive “Tianlong 1” varieties. A comparison of these two varieties led to the detection of the differential expression of many genes associated with the jasmonic acid (JA), brassinolide (BR), calcium, and mitogen-activated protein kinase (MAPK) signaling pathways, in addition to other changes in the expression of stress-related transcription factors (TFs) and proteins (Xuan et al., 2022). Drought-related regulatory genes and pathways have been characterized through comparative transcriptomics in corn (Liu et al., 2020), rice (Lenka et al., 2011), wheat (Niu et al., 2023), millet (Guo et al., 2023), and cotton (Hasan et al., 2019), offering insight into the transcriptional mechanisms that govern the ability of these crops to tolerate drought stress.

Different plant species exhibit differences in their mechanisms of drought tolerance, with some being better equipped to adjust their tolerance for drought and other adverse environmental conditions (Sun et al., 2020). Barley (*Hordeum vulgare* L.) is the fourth most prominent crop in terms of global production and harvesting area (Kovacik et al., 2024). Barley is relatively drought-tolerant and is grown in over 100 countries (Giraldo et al., 2019), and it is thus frequently employed as a model for studies of cereal drought tolerance since it can tolerate these conditions better than wheat or rice. It also has well-established molecular and genetic resources (Dawson et al., 2015). Prior studies have evaluated how barley plants respond to drought conditions. For example, analyses of phytohormone, physiology, and molecular features in the Otis and Baronesse barley varieties, which exhibit distinct drought tolerance, revealed that drought tolerance-related genes were significantly upregulated or specifically induced in drought-tolerant Otis plants relative to their Baronesse counterparts (Harb et al., 2020). Those varieties with distinct tolerance levels and mechanisms can be utilized to develop breeding programs aimed at producing barley varieties that are more resilient and provide better traits amenable to end-use. The commercial Hindmarsh from Australia exhibits a variety of robust agronomic features, including good performance in areas with low rainfall, and it is widely utilized by farmers (Lauer et al., 2017). At present, the mechanistic basis for drought stress response regulation in the Hindmarsh at the molecular level remains poorly understood.

The Tibet barley XZ5 has previously been reported as a drought-tolerant genotype (He et al., 2015). In this study, Hindmarsh was confirmed to exhibit greater drought tolerance as compared to XZ5, enabling the classification of Hindmarsh as a highly drought-tolerant barley variety. A series of comparative transcriptomic analyses of Hindmarsh and XZ5 seedlings under drought conditions was then performed to clarify the molecular mechanisms that account for drought tolerance in Hindmarsh. Under drought conditions, some genes were found to be more highly expressed in the Hindmarsh relative to XZ5 seedlings, including genes encoding hormone-related signaling molecules, ROS-scavenging antioxidants, regulatory TFs, and calcium-dependent protein kinases. Comprehensive efforts to analyze these genes provide new insight into the ability of barley plants to respond to drought stress while providing a series of candidate genes that can help guide the production of varieties with superior drought tolerance in the future.

## 2 Materials and methods

### 2.1 Plant materials

Seeds of the Hindmarsh, two-rowed barley variety originating from Australia, were obtained from National Germplasm Bank of the Chinese Academy of Agricultural Sciences. Seeds of the XZ5, six-rowed barley genotype originating from Tibet, were provided by the College of Agriculture and Biotechnology, of Zhejiang University.

### 2.2 Barley cultivation and drought treatment

Hindmarsh and XZ5 seeds that were full and uniformly plump were sown in 5 L plastic pots (30 × 20 × 17.5 cm) containing an identical substrate mix (peat: perlite: vermiculite = 3:1:1), followed by uniform enrichment with the same amount of slow-release fertilizer. On day 7 of germination, four seedlings were left in each pot. Experiments were performed in a glass greenhouse with respective daytime and nighttime temperatures of 23°C and 20°C, a 16-h light/8-h dark cycle, 50% relative humidity, and a light intensity of 2,500 Lux. All pots were initially watered consistently to maintain ∼80% soil moisture. When seedlings reached the three-leaf stage, drought treatment was initiated by continuing to water the control group but withholding water from the treatment group. A minimum of three biological replicates were included per treatment group. On days 7 and 14 of drought treatment, the aboveground portions of these plants were frozen with liquid nitrogen and stored at −80°C for further analysis. After 14 days of treatment, plant phenotypes were also imaged and evaluated, and the aboveground portions of these plants were collected for measurements of fresh and dry weight.

### 2.3 Library construction and transcriptomic sequencing

The SteadyPure Plant RNA Extraction Kit (Accurate Biology, Qingdao, China) was used as directed to isolate RNA, after which a NanoDrop 2000 (Thermo, Fisher Scientific, Waltham, MA, United States) was used to assess the purity and quality of RNA. Then, Oligo (dT) magnetic beads were used to enrich mRNA which was randomly fragmented into short segments. Reverse transcription was used to prepare cDNA, followed by end repair, A-tailing, and sequencing adapter attachment. Adapter sequences were used to select cDNA fragments, followed by amplification to produce a cDNA sequencing library. This cDNA library was initially quantified with Qubit2.0, with an Agilent 2,100 being used for fragment length analyses, and effective concentrations being quantified by qPCR to ensure appropriate quality.

The Illumina HiSeq 2,500 platform was used for the 2 × 100 bp dual-ended sequencing of the qualified cDNA library to generate raw sequencing reads. These data were initially filtered with Fastp (Chen et al., 2018), removing adapter sequence-containing reads, paired reads with an N ratio >10%, and paired reads with >50% low-quality bases (Q ≤ 20). The resultant clean reads were then aligned to the barley genome (https://ftp.ensemblgenomes.ebi.ac.uk/pub/plants/release-59/fasta/hordeum_vulgare/dna/) and structural annotation files with HISAT2 (Kim et al., 2015), after which the results were subjected to statistical analyses.

### 2.4 DEG identification

Levels of gene expression were quantified with Fragments Per Kilobase of Transcript Per Million Fragments Mapped (FPKM) values, and the alignment of reads was performed with the featureCounts protocol (Liao et al., 2014). DESeq2 was used to analyze differential expression (Love et al., 2014), after which the Benjamini–Hochberg method (Li and Barber, 2019) was employed to correct for multiple hypothesis testing to generate False Discovery Rate (FDR) values. DEGs were then selected with the following criteria: |log_2_fold change|≥1 and FDR <0.05, where fold change denotes the expression ratio of drought to control conditions for a given sample.

### 2.5 Functional enrichment analyses

A BLAST program (E value<10^–5^) was used for the annotation of all DEGs with the NCBI nonredundant (NR), Swiss-Prot, Gene Ontology (GO), euKaryotic Orthologous Groups (KOG), and Kyoto Encyclopedia of Genes and Genomes (KEGG) databases. Those DEGs that were (1) upregulated in Hindmarsh but unchanged or downregulated in XZ5 or (2) unchanged in Hindmarsh but downregulated in XZ5 were selected. GO and KEGG annotation results for these DEGs were analyzed with a hypergeometric test to assess GO and KEGG pathway enrichment (Rivals et al., 2007). The degree of enrichment was quantified as the ratio of the number of DEGs for a particular term to the total number of genes associated with that term.

### 2.6 Mapman and PPI analyses

To better understand the metabolic pathways related to drought tolerance-associated DEGs, the barley genome sequence was submitted to the Mercator Automated Sequence Annotation Pipeline within the MapMan portal (https://mapman.gabipd.org/), thereby generating an appropriate mapping file. The categorization and visualization of drought tolerance-associated DEG-related metabolic pathways were then performed with MapMan v 3.6.0RC1. Interactions among these DEGs were assessed using the STRING tool (https://cn.string-db.org/) to assess protein-protein interactions with the following settings: Organisms: Hordeum vulgare, Network type: full STRING network, Required score: high confidence (>0.7), FDR stringency: medium (5 percent). Gene interactions were visualized with Cytoscape v 3.7.2.

### 2.7 qPCR

Samples of RNA from the Hindmarsh and XZ5 were prepared from potted materials as in Section 4.2. RNA quality was assessed with a NanoDrop 2000 spectrophotometer (Thermo Fisher Scientific, Waltham, MA, United States). cDNA was obtained with an Evo M-MLV Reverse Transcription Kit for qPCR (Accurate Biology, Qingdao, China), while qPCR was performed with a SYBR Green Pro Taq HS Premixed type Kit (Accurate Biology, Qingdao, China) and a ABI QuantStudio™ 3 Real-Time PCR Instrument (Thermo Fisher Scientific, Waltham, MA, United States) as follows: 95°C for 30 s, 40 cycles of (95°C for 5 s and 60°C for 30 s). Melt curves were generated to confirm primer specificity by gradually increasing temperatures from 60°C to 95°C, with a 0.5°C increment at 5 s intervals. Actin was used for normalization purposes, and relative expression was assessed with the -∆∆Ct relative method. All qPCR primers are listed in Supplementary Table S1.

### 2.8 Yeast-based validation of drought tolerance genes

The ORF of the *HvSnRK2* gene (*HORVU.MOREX.r3.1HG0057490*) from Hindmarsh was amplified with a specific primer pair (Supplementary Table S1), with the resultant amplicon then being introduced into the pYES2-NTB vector. The pYES2-NTB-HvSnRK2 (experimental) and pYES2-NTB (negative control) plasmids were then used for the separate transformation of the INVSC1 yeast strain, and sequencing was used to validate appropriate clones. Positive clones were suspended in ddH_2_O, adjusted to an OD of ∼0.2, and a 100 μL volume was used to inoculate 10 mL of SG-Ura liquid medium with a range of PEG 3350 gradients (0, 30, 60, 90, 100, 110, 120, 135 mM). After shaking for 1–2 days at 28°C, the growth status of these samples was assessed. Samples were diluted along a gradient (10^0^, 10^–1^, 10^–2^), spotted onto SG-Ura plates, and incubated at 28°C. Following a further 3- to 4-day growth period, plated colonies were analyzed and imaged.

### 2.9 Statistical analyses

IBM SSPS 22 was used to analyze all data. One-way ANOVAs and least-significant difference (LSD) tests were used to assess differences in dry/fresh weight and gene expression (P<0.05), and the significance of the results was denoted with the Duncan letter notation approach. Significant differences in relative dry weight and fresh weight were analyzed with the Independent samples t-test.

## 3 Results

### 3.1 Hindmarsh is a highly drought-tolerant barley variety

**FIGURE 1:**
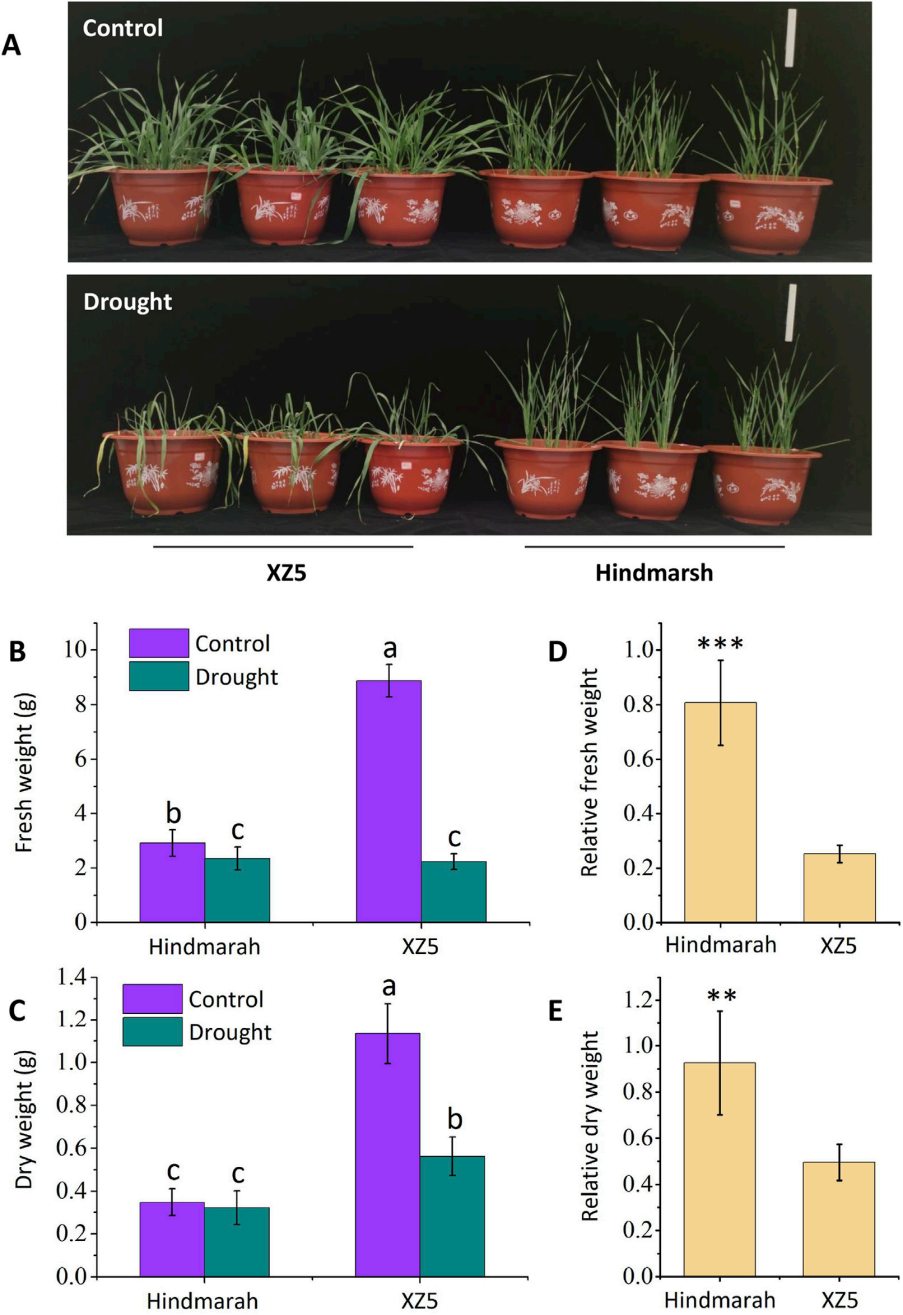
Pot-based experiments were used to evaluate the drought tolerance of the cultivate barley Hindmarsh and wild barley XZ5. **(A)** Phenotypic characteristics of Hindmarsh and XZ5 exposed to control or drought conditions, Bar = 15 cm. **(B, C)** Fresh and dry weights of Hindmarsh and XZ5 under control and drought conditions. **(D, E)** Relative fresh and dry weights of Hindmarsh and XZ5. Different lowercase letters indicates significant differences (*p*<0.05). **p<0.01, ***p<0.001.

The pot-based simulated drought experiment revealed significantly less shoot yellowing and wilting in Hindmarsh compared to XZ5 (Figure 1A). Relative to control plants, XZ5 exhibited significant reductions in shoot fresh weight (Figure 1B) and dry weight (Figure 1C) following drought exposure. While the shoot fresh weight (Figure 1B) of Hindmarsh similarly decreased, there was no corresponding reduction in dry weight (Figure 1C). When impacted by drought, the respective relative fresh weights of Hindmarsh and XZ5 seedlings were 80.7% and 25.2% (Figure 1D), with corresponding relative dry weights were 92.7% and 49.5% (Figure 1E). Hindmarsh exhibited a significantly greater relative biomass than XZ5, emphasizing the superior drought tolerance of Hindmarsh.

### 3.2 Transcriptomic sequencing and identification of drought tolerance-associated DEGs

Next, an Illumina instrument was used for the transcriptomic sequencing of shoots from Hindmarsh and XZ5 seedlings subjected to control conditions or drought for 7 or 14 days. The 24 libraries prepared for this experiment yielded 38,379,664 to 340,897,234 clean reads, with GC ratios ranging from 55.30% to 57.31% and a Q30 range of 90.78%–96.67% (Table 1). Between 92.40% and 94.66% of clean reads were successfully aligned with the reference genome, with 87.27%–90.85% aligning to unique positions, and 3.65%–5.22% aligning to multiple genomic positions. Over 17,000 expressed genes were detected in each sample (Table 1). A total of 68,602 genes were detected across these 24 samples, providing sufficient data for downstream DEG analyses.

**TABLE 1:** Shoot transcriptomic sequencing results for Hindmarsh and XZ5 under control conditions or following drought stress for 7 or 14 days.

Samples	Total reads	GC Content (%)	Q30 (%)	Mapped reads	Uniq mapped	Multiple mapped	‘+’ mapped	‘-’ mapped	Expressed gene Number
H-CK-7-1	50,850,210	56.02	91.45	47,816,067 (94.03%)	45,870,551 (90.21%)	1,945,516 (3.83%)	25,221,960 (49.60%)	25,251,530 (49.66%)	18,141
H-CK-7-2	90,710,864	56.61	90.99	85,416,819 (94.16%)	81,688,569 (90.05%)	3,728,250 (4.11%)	45,286,330 (49.92%)	45,365,244 (50.01%)	18,430
H-CK-7-3	340,897,234	56.44	91.18	320,857,290 (94.12%)	306,193,977 (89.82%)	14,663,313 (4.30%)	170,411,321 (49.99%)	170,710,224 (50.08%)	18,516
H-CK-14-1	74,834,356	56.95	91.54	70,487,006 (94.19%)	67,258,397 (89.88%)	3,228,609 (4.31%)	37,496,240 (50.11%)	37,492,886 (50.10%)	18,721
H-CK-14-2	82,562,342	56.67	91.61	77,890,783 (94.34%)	74,567,603 (90.32%)	3,323,180 (4.03%)	41,250,491 (49.96%)	41,286,989 (50.01%)	18,897
H-CK-14-3	238,023,756	56.55	91.47	223,517,934 (93.91%)	213,074,822 (89.52%)	10,443,112 (4.39%)	119,352,232 (50.14%)	119,431,970 (50.18%)	18,908
H-T-7-1	96,280,970	57.00	91.26	91,141,171 (94.66%)	85,648,359 (88.96%)	5,492,812 (5.70%)	49,606,570 (51.52%)	49,632,103 (51.55%)	17,594
H-T-7-2	104,797,932	56.96	90.78	98,898,720 (94.37%)	93,843,136 (89.55%)	5,055,584 (4.82%)	53,029,499 (50.60%)	53,082,754 (50.65%)	17,296
H-T-7-3	80,819,422	56.82	90.88	75,961,829 (93.99%)	71,833,609 (88.88%)	4,128,220 (5.11%)	40,874,362 (50.57%)	40,958,736 (50.68%)	17,435
H-T-14-1	40,433,202	55.30	96.67	37,361,819 (92.40%)	35,287,588 (87.27%)	2,074,231 (5.13%)	20,212,012 (49.99%)	20,211,821 (49.99%)	18,345
H-T-14-2	91,863,410	56.27	91.39	85,738,458 (93.33%)	80,946,752 (88.12%)	4,791,706 (5.22%)	46,537,351 (50.66%)	46,538,056 (50.66%)	18,833
H-T-14-3	99,338,976	56.70	90.93	92,885,551 (93.50%)	88,013,810 (88.60%)	4,871,741 (4.90%)	50,092,938 (50.43%)	50,119,536 (50.45%)	18,583
W-CK-7-1	115,022,370	56.66	91.47	108,490,526 (94.32%)	103,976,271 (90.40%)	4,514,255 (3.92%)	57,248,791 (49.77%)	57,406,179 (49.91%)	19,225
W-CK-7-2	74,815,382	56.59	91.91	70,790,605 (94.62%)	67,967,895 (90.85%)	2,822,710 (3.77%)	37,245,025 (49.78%)	37,336,688 (49.91%)	19,094
W-CK-7-3	69,991,432	56.71	91.71	66,033,261 (94.34%)	63,449,091 (90.65%)	2,584,170 (3.69%)	34,720,489 (49.61%)	34,799,896 (49.72%)	18,514
W-CK-14-1	97,175,680	56.68	91.22	91,117,433 (93.77%)	87,568,311 (90.11%)	3,549,122 (3.65%)	47,827,700 (49.22%)	47,947,055 (49.34%)	18,902
W-CK-14-2	98,221,750	56.67	91.39	91,891,187 (93.55%)	87,910,674 (89.50%)	3,980,513 (4.05%)	48,683,633 (49.57%)	48,782,373 (49.67%)	19,373
W-CK-14-3	110,109,942	56.71	91.73	103,728,166 (94.20%)	99,324,287 (90.20%)	4,403,879 (4.00%)	54,896,260 (49.86%)	54,983,600 (49.94%)	18,634
W-T-7-1	75,730,826	57.31	91.71	71,518,427 (94.44%)	68,154,889 (90.00%)	3,363,538 (4.44%)	38,042,971 (50.23%)	38,135,056 (50.36%)	17,316
W-T-7-2	84,463,042	57.31	90.97	79,688,669 (94.35%)	76,086,467 (90.08%)	3,602,202 (4.26%)	42,257,079 (50.03%)	42,394,449 (50.19%)	17,620
W-T-7-3	83,858,942	57.21	91.89	79,361,878 (94.64%)	75,406,693 (89.92%)	3,955,185 (4.72%)	42,469,220 (50.64%)	42,572,657 (50.77%)	17,872
W-T-14-1	42,637,538	56.32	94.95	40,050,524 (93.93%)	38,249,105 (89.71%)	1,801,419 (4.22%)	21,294,023 (49.94%)	21,344,554 (50.06%)	19,559
W-T-14-2	40,386,526	56.62	94.95	38,018,978 (94.14%)	36,177,453 (89.58%)	1,841,525 (4.56%)	20,292,576 (50.25%)	20,349,763 (50.39%)	18,899
W-T-14-3	38,379,664	56.41	95.10	36,052,686 (93.94%)	34,324,062 (89.43%)	1,728,624 (4.50%)	19,246,895 (50.15%)	19,288,826 (50.26%)	19,071

Notes: H-CK-7: a 7-day control sample of Hindmarsh, H-CK-14: a 14-day control sample of Hindmarsh, H-T-7: samples of Hindmarsh that underwent drought treatment for 7 days, H-14-7: samples of Hindmarsh that underwent drought treatment for 14 days, W-CK-7: a 7-day control sample of XZ5, W-CK-14: a 14-day control sample of XZ5, W-T-7: samples of XZ5 that underwent drought treatment for 7 days, W-T-14: samples of XZ5 that underwent drought treatment for 14 days.

**FIGURE 2:**
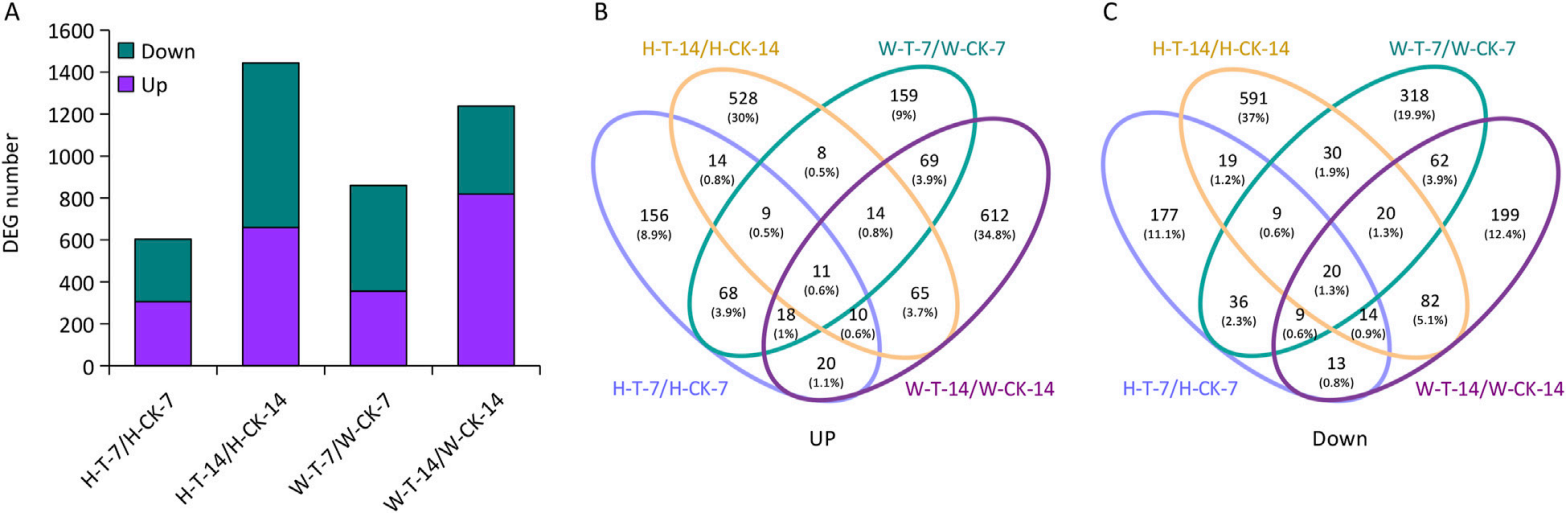
Numbers of identified differentially expressed genes (DEGs) for the H-T-7/H-CK-7, H-T-14/H-CK-14, W-T-7/W-CK-7, and W-T-14/W-CK-14 comparisons. **(A)** A histogram of DEG numbers. **(B, C)** Venn diagrams of up- and downregulated genes. H-T-7/H-CK-7: samples of Hindmarsh that underwent drought treatment for 7 days vs a 7-day control sample of Hindmarsh, H-T-14/H-CK-14: samples of Hindmarsh that underwent drought treatment for 14 days vs a 14-day control sample of Hindmarsh, W-T-7/W-CK-7: samples of XZ5 that underwent drought treatment for 7 days vs a 7-day control sample of XZ5, and W-T-14/W-CK-14: samples of XZ5 that underwent drought treatment for 14 days vs a 14-day control sample of XZ5.

DEGs were identified as those genes with FPKM values that differed sufficiently between the control and drought treatment conditions (|log_2_fold change| ≥ 1, FDR <0.05). Fold-change values were noted as follows for different comparisons: H-T-7/H-CK-7 (samples of Hindmarsh that underwent drought treatment for 7 days vs a 7-day control sample of Hindmarsh), H-T-14/H-CK-14 (samples of Hindmarsh that underwent drought treatment for 14 days vs a 14-day control sample of Hindmarsh), W-T-7/W-CK-7 (samples of XZ5 that underwent drought treatment for 7 days vs a 7-day control sample of XZ5), and W-T-14/W-CK-14 (samples of XZ5 that underwent drought treatment for 14 days vs a 14-day control sample of XZ5). The DEGs for these four comparisons were analyzed (Figure 2A), revealing 603 DEGs for the H-T-7/H-CK-7 comparison of which 306 and 297 were up- and downregulated, respectively (Supplementary Table S2). The H-T-14/H-CK-14 comparison yielded 1,444 DEGs, of which 659 and 785 were up- and downregulated, respectively (Supplementary Table S3). In total, 860 DEGs were identified for the W-T-7/W-CK-7 comparison, of which 356 and 504 were up- and downregulated, respectively (Supplementary Table S4), while 1,238 DEGs were detected for the W-T-14/W-CK-14 comparison, among which 819 and 419 were respectively up- and downregulated (Supplementary Table S5). Further analyses of the shared upregulated (Figure 2B) and downregulated (Figure 2C) DEGs among these four comparisons were next performed, revealing 1,206 drought-related DEGs (Supplementary Table S6). After exposure to drought conditions, these genes were upregulated in Hindmarsh but downregulated or unchanged in XZ5, or were stably expressed in Hindmarsh but downregulated in XZ5.

### 3.3 Go and pathway enrichment analyses

**FIGURE 3:**
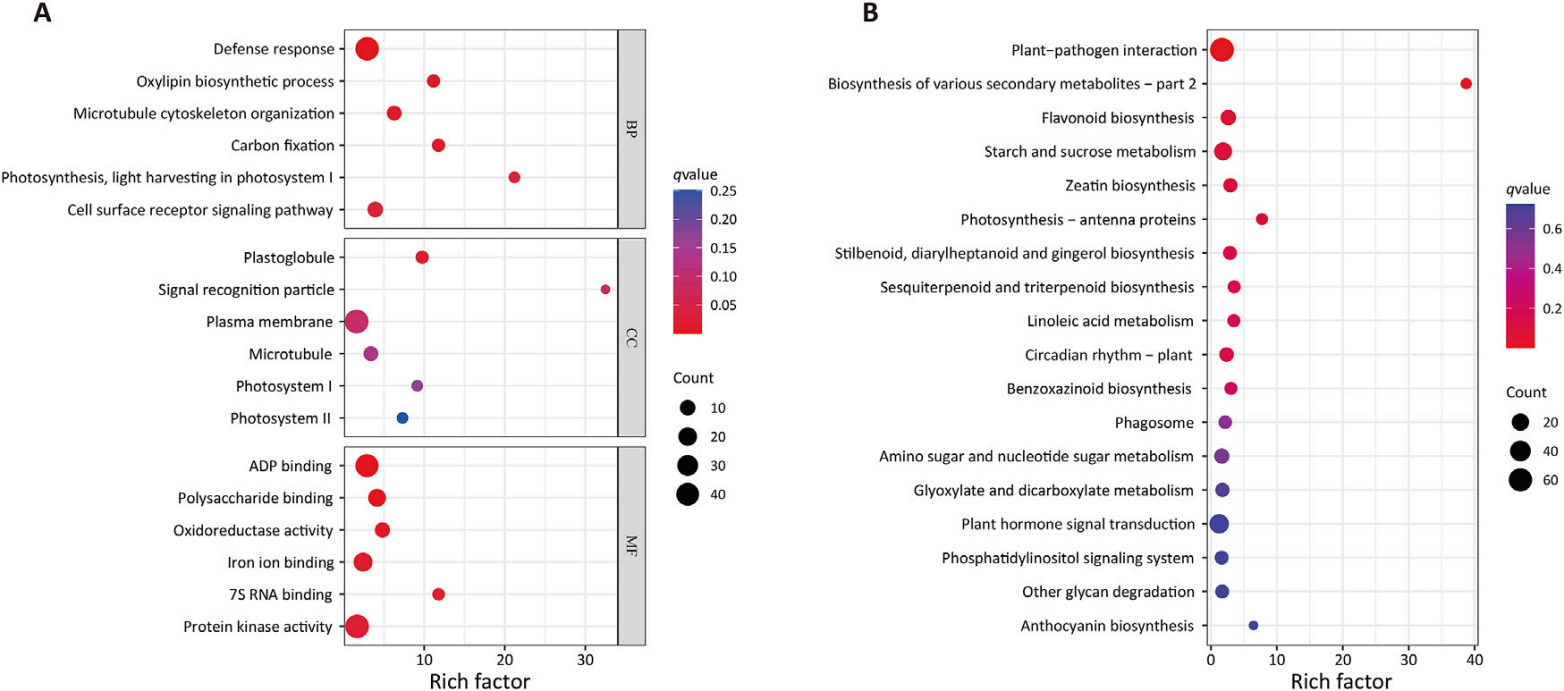
GO and KEGG enrichment analyses of DEGs associated with drought tolerance. **(A)** GO enrichment. BP, biological process, CC, cellular component, MF, molecular function. **(B)** KEGG enrichment. Larger rich factor values are indicative of greater enrichment, while larger dot sizes are indicative of more genes, and redder coloration is indicative of more significant enrichment.

GO enrichment analyses (Figure 3A) of these drought-associated DEGs revealed that the most significantly enriched GO biological process term was Defense response (GO:0006952, q-value = 1.54E-07), with the DEG gene set including 44/638 genes associated with this term. Other enriched GO terms in this category included Microtubule cytoskeleton organization (GO:0000226), Carbon fixation (GO:0015977), Oxylipin biosynthetic process (GO:0031408), Cell surface receptor signaling pathway (GO:0007166) and Photosynthesis, light harvesting in photosystem I (GO:0009768), with respective Rich factors of 6.28, 11.78, 11.16, 3.39, and 21.20. The most enriched cellular component term was Plastoglobule (GO:0010287), with the DEG gene set including 5/25 genes associated with this term. The GO term with the highest Rich factor is signal recognition particle (GO:0048500), with a corresponding Rich factor of 32.5. The top six most enriched GO molecular function terms were ADP binding (GO:0043531), Polysaccharide binding (GO:0043531), Oxidoreductase activity (GO:0016709), Iron ion binding (GO:0005506), 7S RNA binding (GO:0008312), and Protein kinase activity (GO:0004672), with corresponding q-values of 2.84E-07, 0.00, 0.01, 0.02, 0.02, and 0.03, and respective Rich factors of 2.90, 4.15, 4.82, 2.41, 11.79, and 1.67.

KEGG enrichment results (Figure 3B) indicated that these DEGs were significantly enriched in various biosynthesis and metabolic process-related pathways. The Biosynthesis of various secondary metabolites (ko00998) pathway was the most strongly enriched, with a Rich Factor of 38.72. The Plant-pathogen interaction (ko04626) pathway was most significantly enriched, with the DEG gene set including 64/1,484 genes associated with this pathway. Other highly enriched KEGG pathways included the Flavonoid biosynthesis (ko00941), Starch and sucrose metabolism (ko00500), Zeatin biosynthesis (ko00908), Photosynthesis - antenna proteins (ko00196), Stilbenoid, diarylheptanoid and gingerol biosynthesis (ko00945), Sesquiterpenoid and triterpenoid biosynthesis (ko00909), Circadian rhythm–plant (ko04712), Linoleic acid metabolism (ko00591), and Benzoxazinoid biosynthesis (ko00402) pathways.

### 3.4 PPI network-based identification of key drought tolerance proteins

**FIGURE 4:**
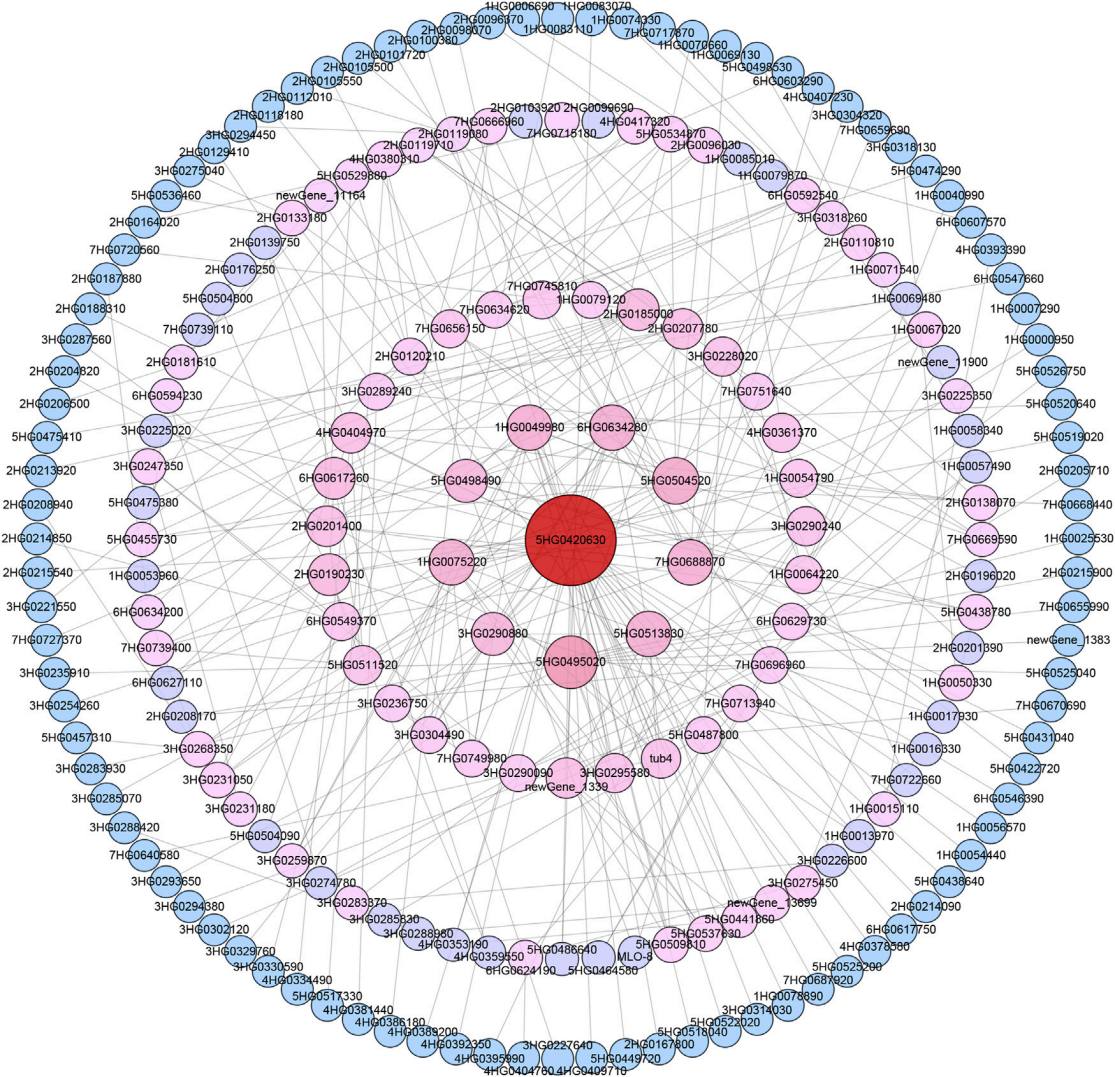
Protein-protein interaction network for drought tolerance-related DEGs. Darker red coloration and larger circle indicates more interactions. The number represents the abbreviated gene ID. The core gene ID is *HORVU. MOREX.r3.5HG0420630*, this gene encodes an Ankyrin protein.

Barley annotation data in the STRING database were then used to establish a PPI network for the proteins encoded by these 1,206 drought tolerance-related DEGs. Interactions were ultimately detected among 368 of these proteins (Figure 4; Supplementary Table S7). The core protein (Ankyrin repeat domain-containing protein 17-like isoform X2) in the established network was encoded by *HORVU. MOREX.r3.5HG0420630*, engaging in interactions with 49 other gene products. Under drought stress conditions, this gene was upregulated in Hindmarsh yet remained unchanged in XZ5. The second tier of interactions in this network included proteins encoded by 9 genes, including *HORVU. MOREX.r3.5HG0495020*, *HORVU. MOREX.r3.5HG0504520*, *HORVU. MOREX.r3.1HG0049980*, *HORVU. MOREX.r3.1HG0075220*, *HORVU. MOREX.r3.5HG0513830*, *HORVU. MOREX.r3.6HG0634280*, *HORVU. MOREX.r3.7HG0688870*, *HORVU. MOREX.r3.3HG0290880*, and *HORVU. MOREX.r3.5HG0498490*, with 18, 13, 12, 12, 12, 12, 12, 10, and 10 pairs of interactions with other proteins in the network, respectively. The functional annotations for these respective genes were as follows: Predicted protein, Phytoene synthase, Actin-2, actin-7, Tubulin alpha-2 chain, Putative RACD protein, Monodehydroasorbate reductase, UDP-arabinose mutase 3, and Predicted protein. Of these genes, 6 were upregulated in Hindmarsh under drought conditions while remaining unchanged in XZ5, whereas 3 were unchanged in Hindmarsh but downregulated in XZ5. In the third interaction tier ring in this PPI network, 31 proteins with 5-9 pairs of interactions with other targets in the network were present. These proteins included Glucose-6-phosphate isomerase 1, MYB family transcription factor, Serine hydroxymethyltransferase 4, Ubiquitin-like protein, and genes of Unknown function. In the fourth tier ring in this PPI network, there were 72 proteins with 2-4 pairs of interactions in the network. These interactions primarily involved targets including Calmodulin-like protein, CBL-interacting protein kinase, Heat shock protein, E3 ubiquitin-protein ligase, Serine-threonine protein kinase, and Predicted protein. The outermost circle in the established network was composed of proteins that interacted with only a single other protein, including a range of kinases, TFs, and antioxidant enzymes.

### 3.5 MapMan analysis of key pathway-related genes

**FIGURE 5:**
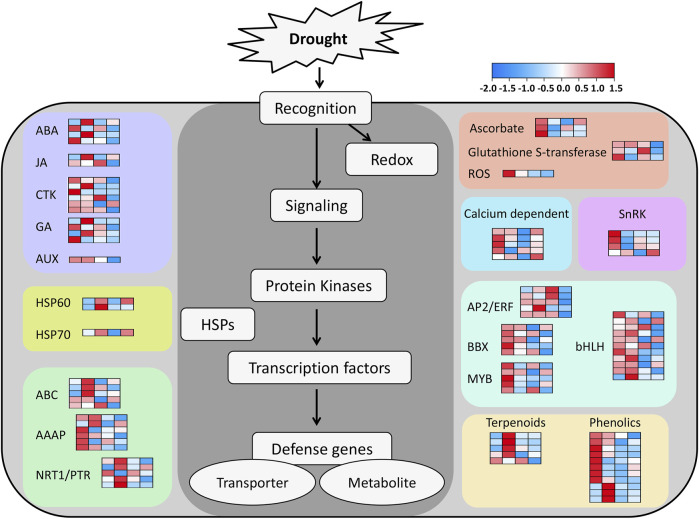
A MapMan pathway-based model for the prediction of drought tolerance in Hindmarsh. The heatmaps are scaled based on Log_2_fold change after row normalization. The four columns in each heat map, from left to right, are as follows: Log_2_H-T-7/H-CK-7, Log_2_H-T-14/H-CK-14, Log_2_W-T-7/W-CK-7, Log_2_W-T-14/W-CK-14. H-T-7/H-CK-7: samples of Hindmarsh that underwent drought treatment for 7 days vs a 7-day control sample of Hindmarsh, H-T-14/H-CK-14: samples of Hindmarsh that underwent drought treatment for 14 days vs a 14-day control sample of Hindmarsh, W-T-7/W-CK-7: samples of XZ5 that underwent drought treatment for 7 days vs a 7-day control sample of XZ5, and W-T-14/W-CK-14: samples of XZ5 that underwent drought treatment for 14 days vs a 14-day control sample of XZ5.

To gain a deeper understanding of the functional categorization of these 1,206 DEGs associated with drought tolerance, the metabolic pathways associated with these genes were explored with MapMan v 3.6.0RC1. Of the 34 pathway types, 373 DEGs across 26 different pathways were detected in this analysis (Supplementary Table S8). The majority of these genes were only located in a single pathway type, but others were involved in two pathway types, as in the case of *HORVU. MOREX.r3.1HG0010450*, which was found to be involved in both the Phytohormone action (Bin code: 11) and Solute transport (Bin code: 24) pathways. Additionally, *HORVU. MOREX.r3.2HG0099690* was found to play a role in both the Cell division (Bin code: 13) and RNA biosynthesis (Bin code: 15) pathways (Supplementary Table S8). Among the 26 analyzed pathways, the greatest number of genes (n = 56) were associated with RNA biosynthesis (Bin code: 15), followed by Protein modification (Bin code: 18) with 50 genes. The remaining pathways were associated with 1–44 genes (Supplementary Table S8). Genes highly expressed in H under drought conditions were identified in the MapMan analysis, highlighting them as putative regulators of the enhanced ability of the Hindmarsh to tolerate drought conditions (Figure 5). These genes primarily consisted of factors associated with signaling, redox activity, protein kinases, TFs, HSPs, and defense-related genes (Supplementary Table S9).

### 3.6 Validation of RNA-seq results

**FIGURE 6:**
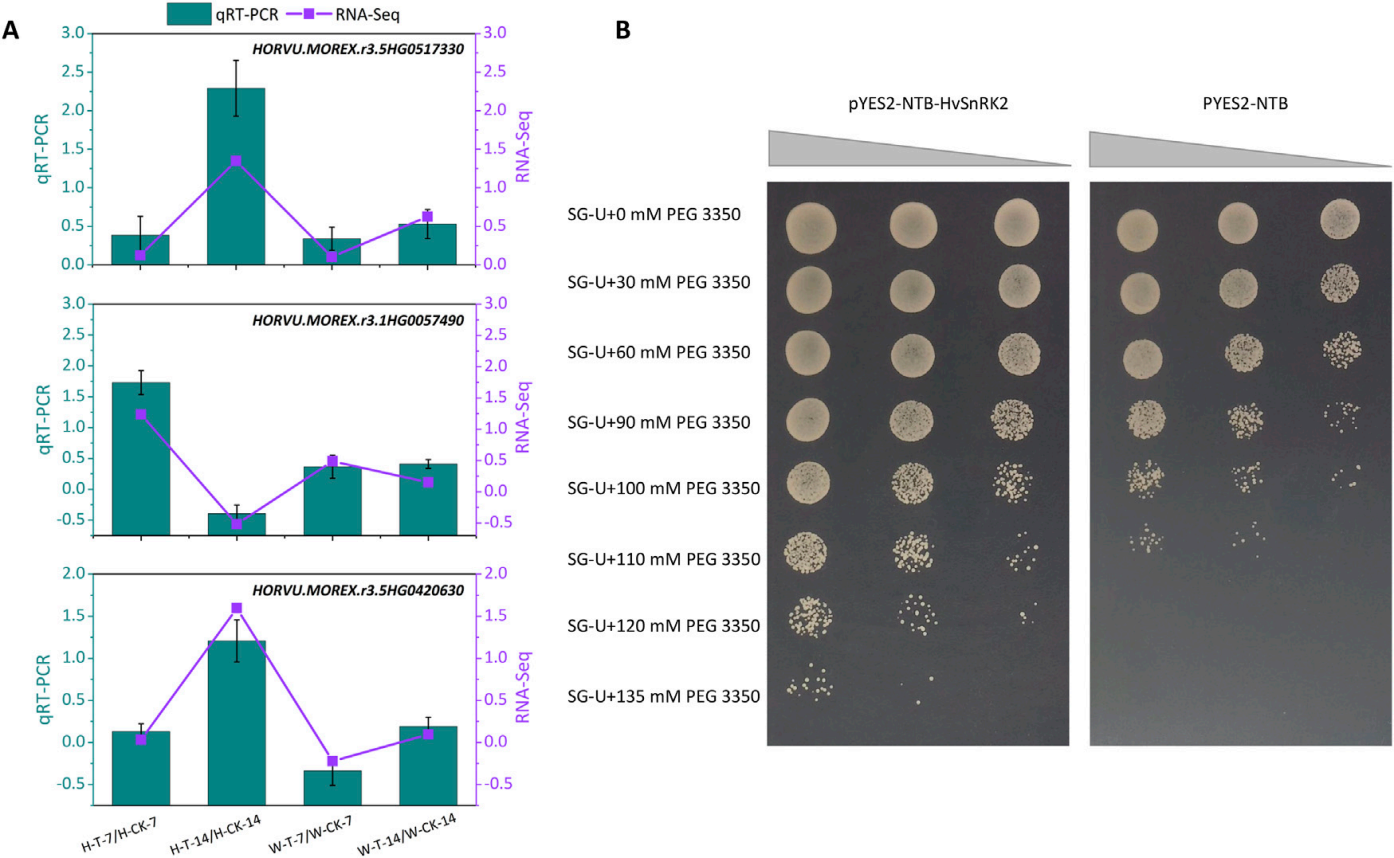
The expression and function validation of some candidate genes in response to drought stress. **(A)** qPCR-based validation of gene expression. qPCR results are presented with a bar chart, with the -ΔΔCt method having been used to assess relative expression. RNA-seq results are presented with a line chart, with log_2_fold change values corresponding to differences in gene expression. H-T-7/H-CK-7: samples of Hindmarsh that underwent drought treatment for 7 days vs a 7-day control sample of Hindmarsh, H-T-14/H-CK-14: samples of Hindmarsh that underwent drought treatment for 14 days vs a 14-day control sample of Hindmarsh, W-T-7/W-CK-7: samples of XZ5 that underwent drought treatment for 7 days vs a 7-day control sample of XZ5, and W-T-14/W-CK-14: samples of XZ5 that underwent drought treatment for 14 days vs a 14-day control sample of XZ5. **(B)** Yeast-based validation of the role of *HvSnRK2* (*HORVU.MOREX.r3.1HG0057490*) in drought tolerance. pYES2-NTB-HvSnRK2 and pYES2-NTB respectively served as the experimental and control groups. Dilution factors from left to right were 10^0^, 10^–1^, and 10^–2^, as represented by the gray triangles.

To confirm the validity of the above RNA-seq and gene screening results, three drought tolerant candidate genes *HORVU. MOREX.r3.5HG0517330* (involving in ABA metabolism), *HORVU. MOREX.r3.1HG0057490* (encoding the SNF1-related protein kinase, HvSnRK2), and *HORVU. MOREX.r3.5HG0420630* (encoding the Ankyrin repeat domain-containing protein 17-like isoform X2) were selected for qPCR analyses. In the transcriptomic dataset, these genes were upregulated in Hindmarsh under drought conditions, but were expressed at a consistent level in W. The qPCR analyses of these genes yielded results consistent with the RNA-seq results (Figure 6A), confirming that these transcriptomic data were sufficiently reliable for further analyses. For instance, the log_2_fold change values for *HORVU. MOREX.r3.5HG0517330* in the H-T-7/H-CK-7, H-T-14/H-CK-14, W-T-7/W-CK-7, and W-T-14/W-CK-14 comparisons were 0.12, 1.35, 0.10, and 0.63, respectively. In qPCR analyses, the respective relative expression levels for this gene were 0.39, 2.29, 0.34, and 0.53. In both analyses, following drought exposure for 14 days, this gene was significantly upregulated in Hindmarsh yet remained unchanged in XZ5.

### 3.7 Validation of *HvSnRK2* as a regulator of drought tolerance

The role of the SNF1-related protein kinase gene *HvSnRK2* (*HORVU.MOREX.r3.1HG0057490*) in drought tolerance was validated through its heterologous expression in yeast (Figure 6B). Under control conditions, yeast with and without *HvSnRK2* expression grew normally on SG-Ura plates. Under drought conditions simulated with a range of PEG 3350 concentrations, however, the growth of yeast expressing *HvSnRK2* was superior to that of control yeast. Increasing PEG 3350 concentrations inhibited yeast growth in a dose-dependent manner, while *HvSnRK2* expression mitigated these effects. At PEG 3350 concentrations of 120 and 145 mM, only those cells expressing *HvSnRK2* were able to grow normally, whereas control yeast growth was fully inhibited. *HvSnRK2* is thus capable of conferring drought tolerance to yeast.

## 4 Discussion

Drought is a prominent abiotic stressor throughout the world, and increasingly severe climate change is contributing to the exacerbation of the negative effects of drought on plant development, agronomic productivity, and overall crop yield and quality (Oguz et al., 2022). Efforts to characterize the mechanisms that enable certain plants to tolerate growth in arid environments and to formulate new approaches to enhancing such resilience are essential to safeguard global food supply in the future (Gupta et al., 2020). Research has demonstrated that XZ5 is a drought-tolerant barley (He et al., 2015). In this study, we demonstrated that Hindmarsh exhibits significantly superior drought tolerance compared to XZ5 (Figure 1). Therefore, exploring new drought-tolerant genes from Hindmarsh holds significant innovation and importance. The crucial drought-tolerant genes identified through comparative transcriptome analysis will ultimately serve crop molecular breeding for drought resistance.

### 4.1 Phytohormones are essential regulators of drought responses in hindmarsh

Phytohormones are essential for the control of plant growth, development, and responses to drought or other environmental stressors. ABA is a key phytohormone that modulates the ability of plants to adapt to water deprivation. Under drought conditions, plants enhance ABA biosynthesis while simultaneously limiting its catabolism. This enhances plants’ resilience under water scarcity, allowing them to endure more effectively (Salvi et al., 2021). ABA can reduce the evaporative loss of water through the control of stomata opening and closing, thereby regulating water retention. Increased ABA concentrations, for instance, can result in the closure of stomata, reduced transpiration-mediated water loss, and better survival in the presence of drought (Liu et al., 2022). ABA can also influence plant root growth and development, driving these roots to absorb more water and extend deeper into the underlying soil (Aslam et al., 2022). Through its ability to activate various drought tolerance-related genes, ABA can also shape the ability of plants to tolerate drought conditions. For example, ABA can reportedly upregulate the expression of *OsbZIP16* and *OsbZIP71*, which are transcription factors that can positively influence resistance to drought in rice plants (Chen et al., 2012; Liu et al., 2014). ABA can also modulate water transport protein activity to influence water uptake and translocation in plants, enhancing their ability to withstand drought exposure (Ding et al., 2016). ABA, together with other phytohormones including auxin (AUX) and cytokinins (CTK), can have synergistic or antagonistic effects on the ability of plants to respond to drought exposure. Synergistic effects of ABA and AUX, for instance, have been shown to bolster drought tolerance in *Arabidopsis thaliana* by controlling gene expression, ROS homeostasis, and root structure (Zhang et al., 2022). Antagonistic interactions between CTK and ABA, in contrast, shape the growth of plants and their adaptation to drought (Huang et al., 2018). In this study, genes associated with phytohormone signaling pathways including the ABA, AUX, and CTK pathways were expressed at higher levels in Hindmarsh relative to XZ5 (Figure 5; Supplementary Table S9). This suggests that these genes play a pivotal role in enhancing the resistance of Hindmarsh to drought conditions owing to their ability to influence hormone metabolism and signal transduction.

### 4.2 Ascorbate and glutathione S-transferase serve as important regulators of the ability of hindmarsh to respond to drought conditions

Ascorbate exhibits several complementary biological functions in plants, including the regulation of exposure to abiotic stressors including drought, extreme temperatures, and increased salinity. As an antioxidant compound, ascorbate can protect plants from ROS-induced oxidative injury, thereby helping to maintain intracellular redox homeostasis (Veljovic-Jovanovic et al., 2017). Together with glutathione, ascorbate is capable of neutralizing ROS including hydrogen peroxide (H_2_O_2_) via the AsA-GSH cycle, thereby preserving the integrity of plant cells (Hasanuzzaman et al., 2019). Increased ascorbate levels in plants have been demonstrated to enhance their ability to tolerate drought conditions. The genetic engineering of plants to increase the ascorbate content therein, for instance, can significantly enhance their growth and survival when exposed to arid environments (Zhang, 2013). Ascorbate is also a key modulator of drought signaling responses in plants, impacting the levels of ABA and other phytohormones to enhance drought tolerance (Wei et al., 2015). Glutathione S transferases are vital for appropriate plant growth, development, and responses to abiotic or biotic stressors. Glutathione S transferases can render plants more resilient under drought conditions through their ability to engage detoxification processes within cells and to help preserve redox homeostasis. *MruGSTU39*, for instance, is upregulated under drought conditions and can facilitate ROS detoxification through increases in the levels of glutathione S transferase and glutathione peroxidase activity, leading to the mitigation of membrane damage and the more robust growth and survival of transgenic *Medicago sativa* (Wang et al., 2022). Relative to wild-type controls, transgenic *Arabidopsis thaliana* plants in which *PeGSTU58* from *Populus euphratica* is overexpressed present with superior antioxidant enzyme activity and greater tolerance for salt and drought stress exposure (Meng et al., 2023). In this study, ascorbate and glutathione S transferase genes were found to be specifically upregulated in the Hindmarsh (Figure 5; Supplementary Table S9). These genes may thus play a key role in the ability of Hindmarsh to resist drought stress through the appropriate control of redox homeostasis under conditions of poor water availability.

### 4.3 HSPs are essential for the preservation of protein integrity and cellular function under drought conditions

Heat Shock Proteins (HSPs) exhibit molecular chaperone functions that help preserve cellular integrity under stressful conditions through the prevention of protein denaturation or aggregation, thereby ensuring appropriate proteostasis and functional integrity. These proteins are thus crucial for the ability of cells to adapt to a range of stressful conditions. HSPs are classified according to their molecular weight, and include HSP100, HSP90, HSP70, HSP60, and several smaller HSPs. The functions and localization of these HSPs vary within cells, but they are all associated with the control of protein folding, assembly, translocation, and degradation (Augustine, 2016). HSP70 is firmly established as a molecular chaperone that can enhance drought tolerance in plants (Aghaie and Tafreshi, 2020). *GhHSP70-26* expression in cotton, for instance, is positively correlated with resistance to drought conditions, and the heterologous overexpression of this gene can endow transgenic tobacco with superior drought tolerance as confirmed by reduced water loss, higher survival rates, and less severe leaf wilting. The overexpression of *GhHSP70-26* in transgenic tobacco also reduced ROS and malondialdehyde (MDA) levels under drought conditions, supporting an active role for this gene as a regulator of drought stress responses through its ability to protect against ROS-induced membrane damage (Ni et al., 2021). The *NtHSP70-8* gene encodes a protein that localizes to the endoplasmic reticulum and is upregulated in response to drought, exogenous ABA, or IAA treatment conditions. *NtHSP70-8* overexpression can significantly induce the upregulation of the ABA synthesis-related *NtNCED3* and *NtNCED5* genes, resulting in elevated ABA concentrations. This coincides with reduced expression of the AUX efflux transporters NtPIN1a, NtPIN1c, and NtPIN3, contributing to increased IAA concentrations and stomatal closure, protecting against transpiration-mediated water loss under drought conditions. Drought stress is also associated with superior antioxidant capacity in plants in which *NtHSP70-8* is overexpressed relative to wild-type tobacco (Song et al., 2021). HSP60 primarily exhibits mitochondrial localization. As an HSP family member, it can be activated and strongly upregulated under abiotic stress conditions whereupon it aids in protein refolding to restore cellular homeostasis. HSP60 may also cooperate with other HSPs to control stress resistance in plant cells (Singh et al., 2019). In this study, genes encoding HSP60 and HSP70 were upregulated in response to drought exposure (Figure 5; Supplementary Table S9), supporting their importance for drought stress adaptation in barley.

### 4.4 SnRKs are essential regulators of adaptation to drought stress in barley

Sucrose non-fermenting-1-related protein kinase (SnRK) is a plant serine/threonine protein kinase that can phosphorylate target substrates to control downstream gene expression, thereby influencing growth, development, and stress responses. The structural features of SnRKs are used to classify them into the SnRK1, SnRK2, and SnRK3 subtypes (Coello et al., 2011). SnRK1 reportedly plays a key role in carbon metabolism and plant development (Wang et al., 2012), whereas the plant-specific SnRK2 and SnRK3 proteins have been linked to the control of signaling pathways responsible for mediating plant responses to osmotic and non-osmotic stressors (Jiang et al., 2022). *OsSAPK8* is a rice gene in the SnRK2 family that can be upregulated in response to drought and other abiotic stressors. The *ossapk8* mutant line was found to exhibit the downregulation of abiotic stress-related marker genes (*OsDREB1*, *OsDREB2*, *OsNCED*, and *OsRAB21*), together with reduced drought tolerance and lower yields as compared to wild-type plants (Zhong et al., 2020). The core ABA signaling pathway consists of three components, including ABA receptors, group A protein phosphatase type 2Cs (PP2Cs), and SnRK2s (Hauser et al., 2017). In the absence of stress, SnRK2 activity is suppressed by PP2Cs which can directly bind or dephosphorylate them to inactivate ABA signaling (Geiger et al., 2009). When exposed to abiotic stress, the synthesis of ABA increases, and ABA is detected by its cognate receptors, with the formation of ABA receptor-ABA-PP2C complexes resulting in PP2C inhibition and SnRK2 release. SnRK2s can then phosphorylate their target proteins, which include ion channels and transcription factors, leading to the increased expression of stress-related genes and ion efflux (Geiger et al., 2009; Umezawa et al., 2009). SnRK3 is also referred to as a calcineurin B-like protein (CBL)-interacting protein kinase (CIPK), and it can transduce stress-related signals through its interactions with CBL, which is a calcium sensor (Albrecht et al., 2003; Sheen, 1996). Here, *SnRK1*, *SnRK2*, and *SnRK3* related genes were all found to be expressed at high levels in Hindmarsh when exposed to drought (Figure 5; Supplementary Table S9). In yeast, heterologous expression assays demonstrated that *HvSnRK2* can enhance drought tolerance (Figure 6B), indicating that these genes may be essential for the regulation of barley drought signaling pathways.

### 4.5 The ANK repeat gene may enhance drought tolerance in barley

Ankyrin repeat (ANK) proteins are members of a family harboring ankyrin repeat sequences together with additional functional domains. This ANK superfamily includes many different functional domain-based subfamilies, which include the ANK-M (ANK repeat sequence motifs only), ANK-TM (transmembrane domain-containing), ANK-TPR (triangular tetrapeptide motif-containing), ANK-RF (RING finger domain), ANK-IQ (calmodulin binding motif), ANK-PK (protein kinase domain), and other subfamilies (Huang et al., 2009). Plants encode many proteins harboring the ANK domain involved in essential processes such as the control of the cell cycle, cytoskeletal interactions, disease resistance, stress responses, and signal transduction (Voronin and Kiseleva, 2008). Drought stress can induce the upregulation of *GmANKTM21*, a member of the ANK-TM family. Relative to wild-type plants, soybean plants overexpressing *GmANKTM21* exhibit higher rates of stomatal closure, lower rates of water loss, a reduction in MDA content, reduced ROS biogenesis, and improved drought tolerance (Zhao et al., 2024). *GmANK114*, a member of the ANK-RF subfamily, is significantly upregulated in response to ABA, drought, and salinity. Relative to wild-type Arabidopsis plants, those transgenic plants overexpressing *GmANK114* present with enhanced germination rates when exposed to drought stress or high levels of salinity. Homologous *GmANK114* overexpression can also increase the survival rates of transgenic soybean hairy roots when exposed to drought and salt stress (Zhao et al., 2020). ANK and ANK-TPR repeat gene clusters have also previously been found to be associated with rice panicle branching diversity (Khong et al., 2021). Relatively little work to date has explored the link between ANK-TPR and drought tolerance. In the present study, an ankyrin repeat domain-containing protein 17-like isoform X2 related gene, which belongs to the ANK-TPR subfamily, was found to be upregulated in Hindmarsh under drought conditions that was centrally located within the established PPI network, interacting with various other proteins (Figure 4; Supplementary Table S7). This gene may thus be a significant regulator of drought tolerance through its ability to interact with other genes.

## 5 Conclusion

Drought is among the most important environmental stressors, imposing a significant agronomic burden owing to reductions in crop yields. Drought-tolerant plant varieties have developed a range of mechanisms through which they can tolerate reduced water availability. In this study, transcriptomic analyses were employed to mine for drought tolerance-related genes in barley using two barley materials with different levels of drought tolerance. Through analyses of the functions, metabolic pathways, and interactions associated with the DEGs that were highly expressed in the more drought-tolerant barley of Hindmarsh, several genes linked to drought tolerance were unveiled. These genes were primarily associated with hormone metabolism, ROS homeostasis, signal transduction, ion transport, transcription factor regulation, and secondary metabolism pathways, suggesting that they may serve as positive regulators of drought tolerance in Hindmarsh. Together, these results offer preliminary insight into the molecular basis for the ability of Hindmarsh to tolerate drought exposure. Further characterization of the mechanisms that govern drought stress responses in Hindmarsh will help further improve crop productivity under arid conditions.

## Data Availability

The original contributions presented in the study are publicly available. This data can be found here: https://www.ncbi.nlm.nih.gov/bioproject/PRJNA1185810.
